# Stephen Massey: a career in visual neuroscience

**DOI:** 10.3389/fopht.2023.1194837

**Published:** 2023-04-25

**Authors:** Stephen L. Mills, David W. Marshak

**Affiliations:** ^1^ Department of Ophthalmology and Visual Science, McGovern Medical School, Houston, TX, United States; ^2^ Department of Neurobiology and Anatomy, McGovern Medical School, Houston, TX, United States

**Keywords:** acetylcholine, glutamate, direction selectivity, gap junctions, cyclic GMP, rods, cones, retina

## Abstract

This review is a memoir by Dr. Stephen C. Massey’s longtime collaborator, Dr. Stephen L. Mills, and written, for the most part, in the first person. It also serves as an introduction to the virtual festschrift to celebrate Dr. Massey’s retirement. and. The references cited here are only a few of the highlights of Dr. Massey’s distinguished career. A complete list is found here: https://pubmed.ncbi.nlm.nih.gov/?term=massey+sc+%28retina+or+photoreceptors%29&sort=date.

## Introduction

This is a remarkable time to study the vertebrate retina, either as a model for the brain or to understand the first steps in vison. We have known about the diversity of retinal neurons and glia for more than one hundred years, and we are now extending these findings and making new discoveries about retinal cell types by analyzing gene expression in single cells. We have made significant progress toward our ultimate goals of describing the neural circuits in the retinas at the level of connections between identified populations of neurons and understanding neuronal and glial cell function at the molecular level. Our colleague at the McGovern Medical School in Houston, Steve Massey, has made many important contributions to this field, both as a researcher and as a mentor, and this Special Topic in Frontiers in Ophthalmology is dedicated to him.

## The eighties

Steve Massey began his scientific career in pharmacology with Mike Neal at the University of London School of Pharmacy and received his Ph.D. in 1974. They developed an *in vivo* in eyecup preparation to study the release of neurotransmitters from from the rabbit retina and used it for the first study of acetylcholine release in response to light stimulation, among other topics (1). Steve then refined these techniques as a postdoctoral fellow with Dianna Redburn (now Johnson) at the University of Texas Health Science Center at Houston, resulting in several seminal papers on the topic (2). I (SLM) would say that Steve’s first major research focus was the study of release and effects of acetylcholine, having written nineteen papers on the subject.

**Figure 1:**
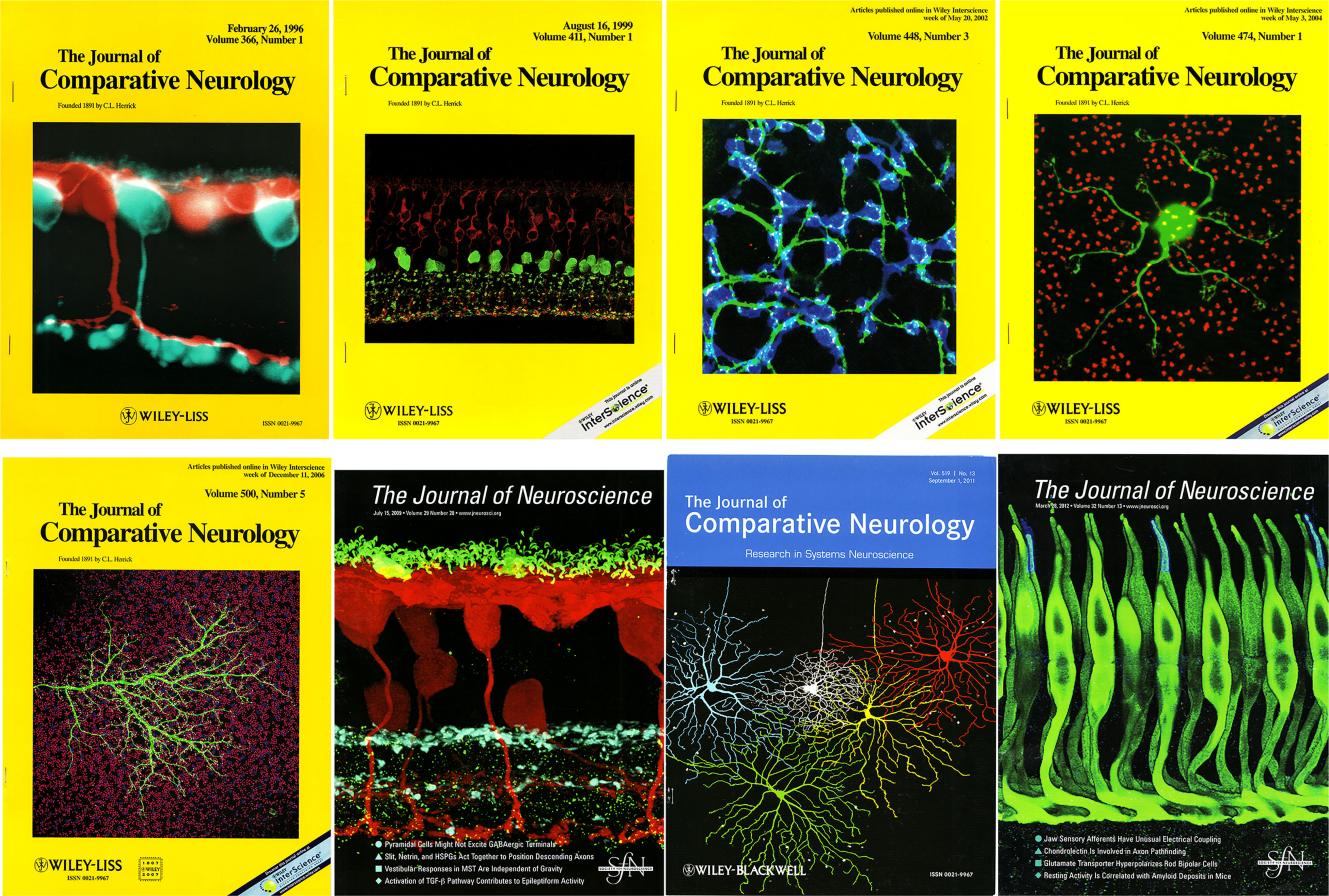
This is a montage of the journal covers that featured images from Steve Massey’s work over the years.

Steve next took a postdoctoral position with Bob Miller at Washington University in Saint Louis, an outstanding lab that had just seen the discovery of the APB receptor by Malcolm Slaughter and Miller. Nigel Daw, Stuart Mangel and Stewart Bloomfield were also working there, among others. Here Steve learned single cell recording, producing several nice papers examining the actions of excitatory amino acids on ganglion cells in the rabbit retina (3). Of course, he later moved on to become a distinguished microscopist at the forefront of exploiting the capabilities of the confocal microscope. **Figure 1** is a montage of his cover illustrations. When I was asked in 1998 to nominate Steve for the next co-chair of the biannual FASEB Summer Conference on Retinal Neurobiology and Visual Processing, he was already well published in pharmacology, physiology, and anatomy. He was awarded the Boycott Prize for achievement in retinal neuroscience at that meeting in 2012.

I first met Steve Massey in 1986, when he returned to Houston to (successfully) interview for an open position at what was then called the Sensory Sciences Center at the University of Texas Health Science Center at Houston (now UTHealth). A recent Ph.D., I was studying color vision psychophysics and beginning to cast about for the next step. Not long after, I was approached by Robert Marc to gauge my interest in doing some work in a funded collaboration with him and Steve, to which I enthusiastically agreed.

Thus began a collaboration that lasted until my retirement in 2020, resulting in 25 papers of which we were joint authors. One of Steve’s abiding interests is in mechanisms of directional selectivity. During his postdoctoral work with Bob Miller, Steve learned the classic tungsten electrode recording techniques of Horace Barlow, Bill Levick, Nigel Daw and others. I initially expected to do this style of blind electrode recording, but he assigned that task to another postdoctoral fellow, Christopher Kittila, with whom he published some nice pharmacological studies on direction selective ganglion cells (4). I was assigned the task of staining starburst amacrine cells targeted with the blue fluorescent dye DAPI and filled with the fluorescent tracer Lucifer Yellow in lightly fixed tissue (5). Initial success was limited, however, and after a few months we switched to live tissue continuously perfused with oxygenated Ames solution. Immediate improvement led to many years of fruitful study.

## The nineties

Much of our joint direction in the 1990s followed from a serendipitous finding one day after the previous day’s intraocular injection of DAPI had resulted in no staining. To rescue the tissue, we incubated the isolated retina in DAPI. After a while some staining appeared and I injected those somas. To our surprise, the stained cells turned out to be not starburst, but AII amacrine cells, which led to the first paper published describing the morphology and distribution of these critical interneurons in rabbit retina (6). We noticed, however, that some other types of somas were also stained, which proved to be A- and B-type horizontal cells and OFF bipolar cells. This of course led to papers describing each of these types. Brian Boycott paid us a couple of visits with his rabbit Golgi slides and was very helpful to us in characterizing the three OFF cone bipolar cell types that we had stained. This period was when I first realized that there was no lab work that Steve Massey loved more than poring over stained retinal neurons in the microscope (to the occasional detriment of the fluorescent samples). Those were exciting times, at the end of a day examining new types of cells as well as staining others previously seen only in Golgi preparations.

About this time, Steve came back from a conference in Australia excited by results shown by Jan de Vente in which increased cGMP stimulation was visualized by an antibody to cGMP that he had developed. We obtained the de Vente antibody, which indicated an increase in cGMP in bipolar cells with appropriate stimulation. We wondered if some modulation of the gap junctional communication between AII amacrine cells and ON cone bipolar cells could be found by application of cGMP modulating drugs. David Vaney and colleagues had recently shown that injection of biocytin into AII amacrine cells could stain not only the injected cell, but also the proximal network of AII and ON cone bipolar cells. They had also demonstrated that dopaminergic stimulation could modulate the AII-AII coupling.

While finishing up another project, I had a go at this idea using the nitric oxide releaser sodium nitroprusside and cGMP analogs as well as the cAMP and dopamine analogs. I had also been investigating on the side to what extent Lucifer Yellow analogs of different sizes diffused through A-type horizontal cell gap junctions. Now that Vaney had discovered the wonderfully successful method of staining electrically-coupled cells with the biocytin-gap junction method, I compared the staining of Neurobiotin (a positively charged analog of biocytin) with a larger relative called biotin-X cadaverine. David Marshak had asked me to present a seminar to his department at this time. Steve had been away for a while and had come back just in time for my presentation. There for the first time he heard the success of his cGMP ideas and the new size-selectivity data. Excitedly, he took the Molecular Probes catalog home to peruse and by the next morning had identified a series of probes for analyzing these interactions. (We had a few synthesized to round out the series.) Thus was laid the groundwork for a paper in Nature that described the cGMP sensitivity of AII amacrine cell/ON cone bipolar cell gap junctions and the implication that multiple connexins might be involved due to size selectivity differences (7). This all occurred not long after nitric oxide was named “Molecule of the Year” in Science magazine. It also led a paper in Journal of Neuroscience using the size series to compare differential gap junctional permeability in the two horizontal cell types and AII and ON cone bipolar cells.

It was about this point, shortly after the Nature paper, that the interdepartmental Vision group at UT-Houston acquired a confocal microscope through a NIH core grant. The core group leader was Dianna Johnson, who had served as Steve’s postdoctoral mentor in the early 1980s. By chance one evening, I was the first person in our group to examine data on the confocal - the staining was in rabbit tissue and produced by an antibody to the glutamate transporter GLT-1. This was a preparation where the total staining resulting from different sources of GLT-1 creates a background sufficiently intense that no single cell was able to be properly observed on a conventional fluorescent microscope. On the confocal, however, a distinct population of one bipolar cell type was clearly evident (among other cell types). My first thought on seeing this result was prophetic, i.e., when Steve Massey sees this, we will never be able to tear him away from the confocal microscope. Steve quickly developed a skill for finely adjusting the several parameters for obtaining outstanding images, which was further enhanced in immunolabeled tissue with a penchant for searching for particularly revealing and aesthetically pleasing locations in the tissue.

## The aughts

Around this time, Steve undertook his last experiment on acetylcholine release from the rabbit retina. Sally Firth, a postdoctoral fellow from Australia, had joined David Marshak’s laboratory to study the role of dopamine in the etiology of myopia. Because exposure to bright sunlight and dopamine both seemed to prevent the development of myopia, she wanted to be the first to describe the release of dopamine from a mammalian retina in response to a wide range of light intensities. But first Sally needed to learn how to prepare the rabbit retina from Steve, and they decided to use the highly selective agonists and antagonists that had been developed recently to characterize the glutamate receptors of cholinergic neurons. They showed that these were AMPA receptors, whose rapid desensitization kinetics enhance the sensitivity of cholinergic neurons to moving and other rapidly changing stimuli (8). In addition to teaching Sally his technique, Steve also contributed some intracellularly-injected cholinergic amacrine cells and identified the receptor subunits that they expressed by confocal microscopy. Soon afterwards, Sally was able to do the experiments that she had planned. With help from David Marshak, Margaret Rice, Laura Frishman and myself, she showed that dopamine release increased monotonically with light intensity, a phenomenon that we now know is mediated by intrinsically-photosensitive retinal ganglion cells.

By this point, Steve’s interest in gap junctions had developed into a major focus, and I was an independent faculty member. In conjunction with John O’Brien, who was the first to identify what is now the connexin36 family and had joined our group, we identified the major patterns of Cx36 staining in the rabbit retina (9). This was excellent work done primarily by a M.D./Ph.D. student in the Massey lab, Jennifer J. O’Brien, who also showed with Steve and John O’Brien that A-type horizontal cells in the rabbit retina used Cx50 for their gap junctions (10). Steve’s and my interests began to diverge somewhat as I continued my interest in characterizing retinal cell types.

Steve was fortunate at this time in taking on a new graduate student named Wei Li, now at NIH and whose indefatigable efforts stimulated that lab broadly over the time he was there. Wei was one of those individuals with a talent for making experiments work – not only his, but catalytically across the lab. Brady Trexler, a talented postdoctoral fellow who had trained in gap junctional recording at Albert Einstein College of Medicine also joined Steve’s lab. This group produced a number of fine papers performing close analysis of retinal circuitry contacts at the confocal level, often involving gap junctions. Over this period, Steve created a number of analytic procedures for quantifying the extent of relationships between processes viewed at the confocal level, such as comparing number of contacts between cell processes when viewed in the proper orientation versus when the channel of one marker has been rotated, thus establishing the chance contact baseline.

One surprising and gratifying result came to us via Hideo Hoshi, an excellent postdoctoral fellow in my lab. After Roska and Werblin (11), we had rediscovered the bistratified diving ganglion cell of the rabbit retina, a purely ON-type cell physiologically, which left us puzzled as to the function of the OFF arbor. Steve and Hideo both often worked evening hours in the confocal room on separate projects and began to recognize in Hideo’s images a heretofore-unknown pathway whereby the OFF dendrites of these cells received en passant gap junctional input from ON bipolar cell axons as they descended through the OFF sublamina, a pattern of bipolar cell contacts we also found on dopaminergic amacrine cells and some ipRGCs (12). David Berson’s lab simultaneously and independently discovered the same phenomenon in mouse retina (13).

## The twenty tens and twenties

Steve had never lost interest in direction selective retinal ganglion cells, and he collaborated with David Marshak again to search for their homologs in primate retinas. The neuropeptide CART had been localized in direction-selective retinal ganglion cells of mice, and so they used antibodies to CART to label baboon retinas. Instead of retinal ganglion cells, they labeled amacrine cells in the primary rod pathway, cells that Steve had studied earlier in the rabbit retina. Steve and Chris Whitaker also contributed intracellularly injected amacrine cells, which made it possible to see individual cells rather than a network of labeled dendrites (14).

In recent years, Steve has undertaken fruitful collaborations with colleagues Christophe Ribelayga, and Chai-An Mao. Steve and Christophe and other essential contributors have recently resolved a number of long-standing mysteries about the nature of rod-cone communication and how it evolves in the 3 rod pathways. This has resulted in a series of superb papers. In collaboration with Christophe Ribelayga and Nange Jin from the Ribelayga lab, David Marshak and others, transgenic mice were used to perform genetic manipulation of rod/cone coupling, thereby revealing the relative contributions of the three rod pathways to the retinal output to an unprecedented degree (15). This study included ultrastructural evidence for these findings that further established that rod-cone coupling mediated by Cx36 is the dominant pathway in the mouse retina, with rod-rod and cone-cone gap junctions being relatively minor or absent. In another study, Cx36-mediated coupling present between rods and cones and their relative contributions to the three rod pathways was delicately teased out using connexin 36 knockout mouse models (16).

In related work, another paper established the anatomical substrates of the tertiary rod to OFF cone bipolar cell pathway in the rabbit retina (17). Jin, Massey, and Ribelayga also contributed to a paper establishing the relative roles of the primary and secondary pathways in mouse in driving non-image forming behaviors in the mouse (18). Finally, using both confocal microscopy and ultrastructural analysis of the outer plexiform layer, the Massey lab and collaborators from the Catherine Morgans, Josh Singer, and Wei Li labs further established the dominance of rod-cone coupling in the mouse retina, including estimates of number of active connexins, their conductance, and the effects of dopamine in modulating this pathway (19). Together, these recent articles are a magnificent coda to a decades-long interest in establishing the role of gap junctions of photoreceptors in the transmission on the visual signal via its various pathways and complement earlier work on Cx36 in AII amacrine cells in the primary pathway.

In addition to his primary research, Steve has provided seminal leadership to the local and national scientific communities in many ways. In 1996, he was chosen to head the UT Houston Vision Research Consortium, as part of a reorganization of the major emphasis on vision research at our institution, as well as other local institutions. In this role, he led efforts that resulted in substantial research funding, serving as PI on an NIH Vision Core Grant, which he maintained for 30 years, and on a long-standing NIH training grant shared with the University of Houston College of Optometry. He was also pivotal in obtaining institutional support from private funding groups, including Research to Prevent Blindness and a strong group of local donors. Additionally, he was organizer of the FASEB meeting on retinal circuitry in 2002, served on several ARVO organizing committees and was a NEI grant reviewer for many years, as a permanent member in addition to a great number of *ad hoc* stints.

## Conclusion

Steve Massey began as a pharmacologist, then trained as an electrophysiologist, and later became an extraordinary neuroanatomist. He continually learned new techniques and then refined them, never losing sight of the fundamental questions. This approach enabled him to make important contributions to visual neuroscience throughout his career.

## Author contributions

SM wrote the first draft of the review. DM edited the manuscript and added additional material. All authors contributed to the article and approved the submitted version.
